# Draft Genome Sequence of Alkane-Degrading *Acinetobacter venetianus* JKSF02, Isolated from Contaminated Sediment of the San Jacinto River in Houston, Texas

**DOI:** 10.1128/genomeA.00286-16

**Published:** 2016-04-14

**Authors:** Rupa Iyer, Ashish Damania

**Affiliations:** a Center for Life Sciences Technology, College of Technology, University of Houston, Houston, Texas, USA; b Department of Pediatrics, Section of Pediatric Tropical Medicine, Baylor College of Medicine, Houston, Texas, USA

## Abstract

*Acinetobacter venetianus* JKSF02 was isolated from contaminated sediment in eastern Houston, Texas along the San Jacinto River. This microorganism specializes in n-alkane degradation and is well suited for bioremediation of the petroleum hydrocarbon deposited throughout the region by shipping and industrial activity from the Houston Ship Channel.

## GENOME ANNOUNCEMENT

The San Jacinto River in eastern Harris County in Houston, Texas is heavily polluted with numerous xenobiotics of concern. Toxic halogenated dioxin and dibenzofuran waste has leaked from two dioxin waste pits under the 1 to 10 overpass for several decades resulting in widespread contamination across the river system (1). In addition, halogenated biphenyls, petroleum hydrocarbons, and other polycyclic aromatics are also present due to the confluence of the river with the Houston Ship Channel and its associated shipping and industrial base to the south. Here, we report a draft genome sequence of a microorganism isolated from contaminated sediment from Diamond Head Island near the site of the San Jacinto waste pits that has been identified as a novel strain of *Acientobacter venetianus*, designated *A. venetianus* JKSF02. *A. venetianus* is a Gram-negative, oxidase-negative bacterium that was initially characterized for notable phosphotriesterase activity against organophosphate insecticides as part of an Environmental Sampling Research Module undertaken by University of Houston biotechnology undergraduates (2). Sequence analysis reveals that JKSF02 is closely related to the oil degrading *A. venetianus* RAG-1^T^ (3) and harbors both alkane and aromatic degradation pathways. In addition, the presence of dioxygenase and ring hydroxylase genes complements this strain’s potential application for remediating the highly recalcitrant organic waste found across the region. The genome sequencing of JKSF02 was performed through Illumina MiSeq paired-end sequencing with a final sequencing coverage of 410×. Sequence reads were checked for quality using Fastqc (http://www.bioinformatics.babraham.ac.uk/projects/fastqc/) and filtered using BBTools (http://sourceforge.net/projects/bbmap). Paired-end reads were then assembled into a total of 87 contigs with the Spades 3.6.2 program (4). Preliminary reference based annotation using Patric (5) was carried out to identify conserved pathways. Final *de novo* annotation was performed with Prokka (6) and the NCBI Prokaryotic Genomes Automatic Annotation Pipeline (http://www.ncbi.nlm.nih.gov/genome/annotation_prok/). The metabolic pathways of aromatic and heterocyclic compounds were examined through KEGG databases (7). This draft genome of strain JKSF02 consists of 3,475,003 bp in length with one known partial plasmid sequence. The JKSF02 genome encodes for 3,148 putative coding sequences, of which 2,314 are predicted to form functional proteins. The genome also contains 8 rRNA, 66 tRNA, and 4 noncoding RNA (ncRNA) loci and harbors 1 clustered regularly interspaced short palindromic repeats (CRISPR) array.

### Nucleotide sequence accession numbers.

The *A. venetianus* JKSF02 whole-genome shotgun (WGS) project has the project accession no. LSVC00000000. This version of the project (01) is LSVC01000000, and consists of sequences LSVC01000001 to LSVC01000087.
